# Enhanced accuracy and sensitivity in detecting *FMR1* CGG repeats: a multicenter evaluation of a novel PCR-capillary electrophoresis assay

**DOI:** 10.1007/s12519-025-00977-5

**Published:** 2025-09-12

**Authors:** Xin-Yi Shou, Zhi-Wei Zhu, Hua Jin, Ji-Hong Hu, Ti-Zhen Yan, Qing-Yan Zhong, Wen-Hao Li, Jian-Hua Mao, Min-Yue Dong, Qiong Xu, Shao-Qing Ni

**Affiliations:** 1 Children’s Hospital, Zhejiang University School of Medicine, National Clinical Research Center for Child Health, National Children’s Regional Medical Center, Hangzhou, China; 2 Department of Developmental and Behavioral Pediatrics, Children’s Hospital, Zhejiang University School of Medicine, National Clinical Research Center for Child Health, National Children’s Regional Medical Center, Hangzhou, China; 3 Department of Prenatal Diagnosis, Jinan Maternal and Child Health Hospital, Jinan, China; 4 Department of Rehabilitation, Hunan Children’s Hospital, Hunan, China; 5 Institute of Reproductive Genetics, Dongguan Maternal and Child Health Hospital, Dongguan, China; 6 Department of Medical Genetics, Liuzhou Matermal and Child Health Care Hospital, Guangxi, China; 7 Department of Nephrology, Children’s Hospital, Zhejiang University School of Medicine, National Clinical Research Center for Child Health, National Children’s Regional Medical Center & Liangzhu Laboratory, Zhejiang University School of Medicine, No. 3333, Binsheng Road, Binjiang District, Hangzhou, China; 8 Department of Reproductive Genetics, Women’s Hospital, Zhejiang University School of Medicine, Hangzhou, China; 9 Department of Child Health Care, Children’s Hospital of Fudan University, Shanghai, China; 10 Clinical Trial Institute, Children’s Hospital of Zhejiang University School of Medicine, National Clinical Research Center for Child Health, National Children’s Regional Medical Center, No. 3333, Binsheng Road, Binjiang District, Hangzhou, China; 11 Research Center for Clinical Pharmacy, College of Pharmaceutical Sciences, Zhejiang University, Hangzhou, China

**Keywords:** Capillary electrophoresis, Diagnosis, FMR1, Fragile X syndrome, Genetic counseling, Trinucleotide repeat expansion

## Abstract

**Background:**

Fragile X syndrome (FXS) is primarily caused by the expansion of CGG repeats in the 5’ untranslated region of the *FMR1* gene. Accurate detection of expanded *FMR1* alleles is essential for timely diagnosis and management. Triplet-repeat primed PCR is the most widely used method for detecting FXS; however, it has limitations in detecting low DNA input (< 10 ng/μL) and low-level mosaicism (< 5%). This study aimed to develop an improved method for detecting *FMR1* CGG repeat expansions, outperforming existing methods in efficiency, reliability and sensitivity.

**Methods:**

We developed a novel four-primer PCR with capillary electrophoresis assay (FP-PCR/CE) and validated its performance in identifying and sizing *FMR1* alleles using DNA standards and multi-center clinical samples (*N* = 1690). Comparative analyses were performed against the AmplideX *FMR1* PCR/CE assay and Southern blot to assess the accuracy, sensitivity, and clinical reliability of this assay.

**Results:**

The FP-PCR/CE assay demonstrated 100% concordance with DNA standards for CGG repeat sizing and mosaicism detection. It detected DNA input ≥ 2.5 ng/μL and mosaic alleles at a mass fraction as low as 1%. In clinical validation, FP-PCR/CE achieved 100% concordance in *FMR1* allele characterization with both the AmplideX assay and Southern blot, while exhibiting higher sensitivity for detecting mosaicism. Additionally, the assay identified AGG interruptions within *FMR1* alleles. The FP-PCR/CE assay also reduced testing time to under 7 h and lowered the cost to < $80 per test.

**Conclusions:**

The FP-PCR/CE assay is a rapid, accurate, and cost-effective method for *FMR1* CGG repeat analysis, offering improved sensitivity for mosaicism detection. Its scalability and reliability support its potential for broader use in FXS carrier screening, clinical diagnosis and research.

**Graphical abstract:**

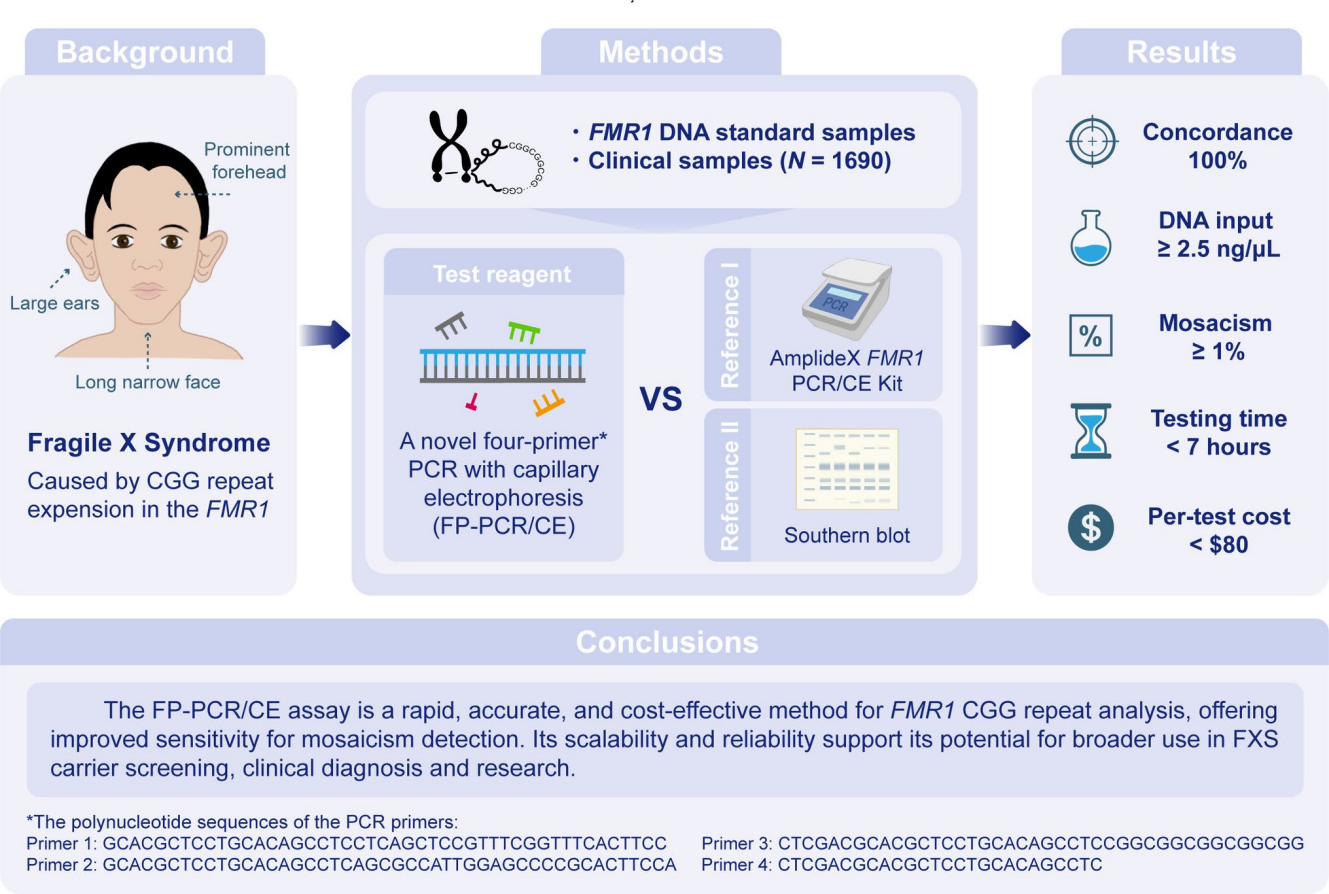

**Supplementary Information:**

The online version contains supplementary material available at 10.1007/s12519-025-00977-5.

## Introduction

Fragile X syndrome (FXS) is the leading inherited cause of intellectual disability and the most commonly known single-gene cause of autism spectrum disorders (ASD) [1, 2], which affects approximately 1 in 4000 males and 1 in 5000–8000 females [3]. FXS is caused by an abnormal CGG trinucleotide repeat expansion and associated aberrant methylation in the 5’ untranslated region (5’ UTR) of the *FMR1* gene (OMIM 309550) located on the X chromosome [4]. The normal (NOR) *FMR1* allele contains 5–44 CGG repeats. Alleles with 45–54 repeats are classified as intermediate (IM), which typically do not cause any symptoms. Premutation (PM) alleles, defined by 55–200 CGG repeats [5], do not cause FXS, but may develop fragile X-associated primary ovarian insufficiency or fragile X-associated tremor/ataxia syndrome [6–8]. Population studies estimate that about 1 in 250–850 males and 1 in 110–300 females carry a PM allele [9], who are at risk for associated conditions and for transmitting a full mutation to their offspring. Full mutation (FM) alleles contain more than 200 CGG repeats and are associated with aberrant hypermethylation, leading to silencing of the *FMR1* gene, loss of the encoded protein FMR1, and FXS [10]. FXS patients typically have intellectual disability, developmental delays, language communication disorders and behavioral issues. This condition imposes a substantial lifelong socioeconomic burden, with direct medical expenses averaging $33,000 per patient annually, and places considerable strain on healthcare systems and familial caregivers [11].

Since there are currently no specific disease-modifying therapies for FXS [12–14], the early identification of fragile X premutation carriers and genetic counseling is critical to reduce the incidence of FXS [15, 16]. Current molecular diagnostic methods include Southern blotting, polymerase chain reaction (PCR), and emerging long-read sequencing technologies [17]. Southern blotting used to be considered the gold standard for detecting FM alleles and analyzing *FMR1* methylation; however, it is time-consuming requiring a large amount of DNA, and lacks precision in sizing CGG repeats [17]. Conventional PCR offers a faster turnaround time but is limited in amplifying GC-rich regions of *FMR1*, often failing to detect FM alleles [18]. While long-read sequencing provides high accuracy, its routine clinical use is constrained by high costs and complex data analysis [19]. Triplet-repeat primed PCR (TP-PCR), which utilizes CGG-specific primers, improves upon conventional PCR and is now the leading method for *FMR1* testing [17, 20]. One example is the AmplideX *FMR1* PCR/CE Kit, the first *FMR1* gene testing reagent approved by the U.S. Food and Drug Administration (FDA). AmplideX reliably amplifies alleles with > 1000 CGG repeats and can detect PM and FM alleles, including mosaic and homozygous cases. Despite its advantages, AmplideX still has technical limitations, including reduced sensitivity at low DNA input concentration (below 10 ng/μL) and a limited ability to detect low-level mosaicism (typically < 5%) [21–23]. These constraints may increase the risk of missed and delayed diagnoses, increased emotional and financial burden, and missed opportunities for timely genetic counseling and early intervention.

To address these limitations in *FMR1* testing, we used a novel *FMR1* assay that combines four-primer PCR with capillary electrophoresis (CE) for detection. In this study, we evaluated the performance of this assay as a more sensitive, efficient, and practical diagnostic tool for FXS.

## Methods

### Study design

The four-primer *FMR1* PCR/capillary electrophoresis assay (FP-PCR/CE) was developed by Xiamen Biofast Biotechnology Co., Ltd. (U.S. Patent No. US11459614B2; Chinese Patent No. 2019105488480) was evaluated as the test reagent. The AmplideX PCR/CE *FMR1* Reagents kit (AmplideX) from Asuragen, Inc. was used as reference reagent I, and Southern blot was used as reference reagent II.

**Fig. 1:**
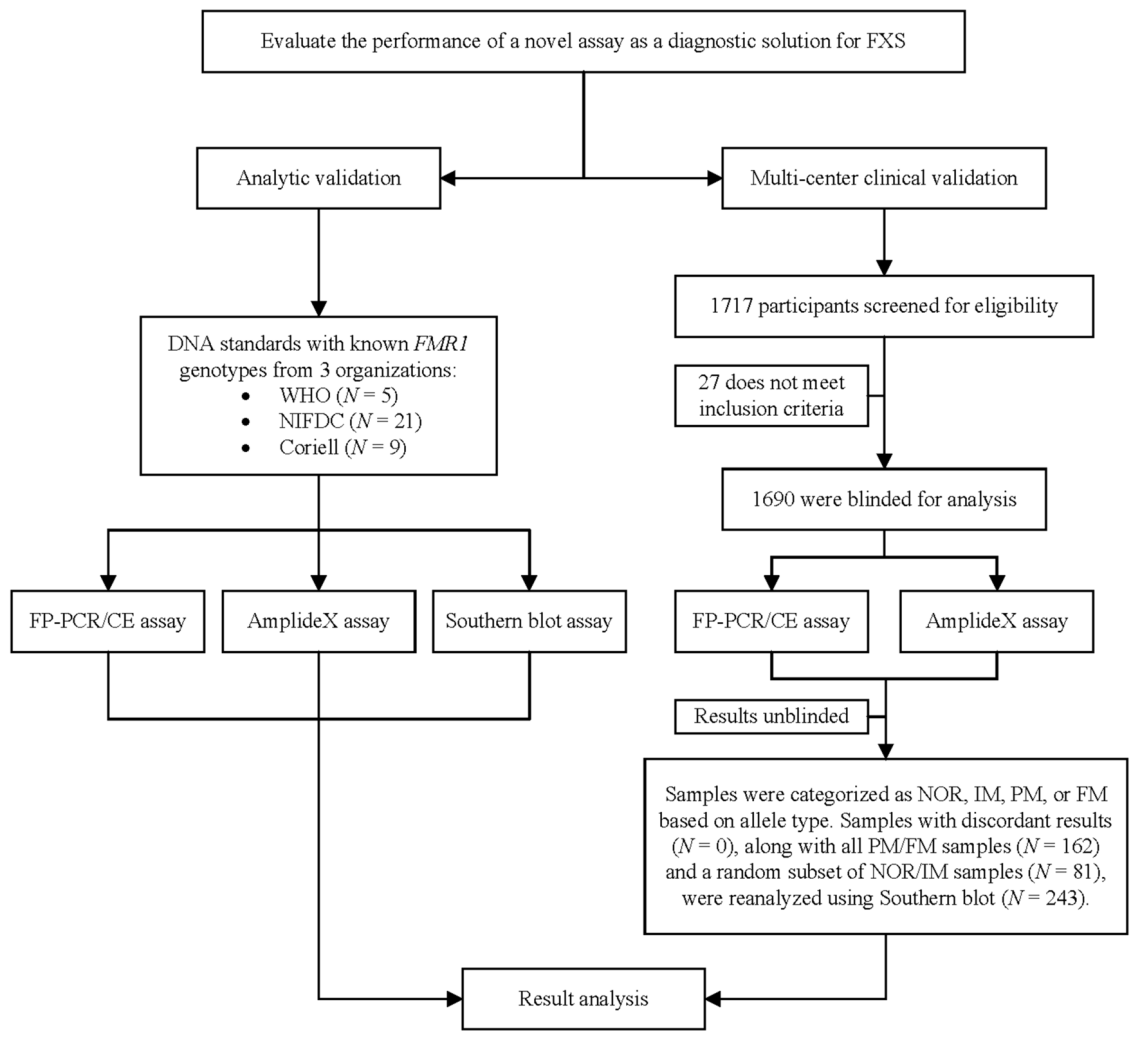
Study flowchart. *FXS* fragile X syndrome, *WHO* World Health Organization, *NIFDC* National Institute of Food and Drug Control, *Coriell* Coriell Institute for Medical Research, *FP-PCR/CE* four-primer *FMR1* PCR/capillary electrophoresis assay, *NOR* normal, *IM* intermediate, *PM* premutation, *FM* full mutation

DNA standards were used to validate the analytic performance of FP-PCR/CE. A multi-center study was performed to evaluate the clinical adoption of FP-PCR/CE. During the study, samples were blinded and tested using both FP-PCR and the AmplideX assay. After testing, the samples were unblinded and categorized as NOR, IM, PM, or FM based on allele type. Samples with discordant results, along with all PM/FM samples and a random subset of NOR/IM samples, were reanalyzed using Southern blot (Fig. 1).

### Standard samples

DNA standards with known *FMR1* genotypes from three organizations: (1) World Health Organization (WHO): 07/120 (NOR), 07/122 (PM), 07/174 (PM), 07/168 (FM), and 07/170 (FM); (2) National Institute of Food and Drug Control (NIFDC): N1–N6 (NOR), N7–N8 (non-human DNA), N9 (*FMR1* deletion), P1–P2 (IM), P3–P6 (PM), P7–P10 (FM), and P11–P12 (size mosaic); (3) Coriell Institute for Medical Research (Coriell): NA18073 (NOR female), NA13537 (NOR male), GM20230/NA20232 (IM male), NA06894/NA06905/NA06968 (PM female), GM06891/NA06892 (PM male), GM07537 (FM female), NA04025/NA06897 (FM male).

### Clinical sample collection

EDTA-anticoagulated whole blood clinical samples were collected according to the inclusion and exclusion criteria (detailed below) from six centers in China: Children’s Hospital, Zhejiang University School of Medicine; Women’s Hospital, Zhejiang University School of Medicine; Jinan Maternity and Child Care Hospital; Liuzhou Maternity and Child Healthcare Hospital; Children’s Hospital of Fudan University; and Hunan Children’s Hospital. In this study, positive blood samples (previously characterized as FM) could be retrospectively enrolled. All enrolled patients provided written informed consent for molecular analyses prior to the collection of tissue specimens. The use of residual EDTA-anticoagulated peripheral blood from previous clinical tests was exempt from informed consent. Samples were stored short-term (up to 2 weeks at 2–8 °C), intermediate-term (up to 2 years at − 20 ± 5 °C), or long-term (up to 5 years at − 70 °C), with a maximum of four freeze–thaw cycles. Genomic DNA was extracted using the Biofast Nucleic Acid Extraction Kit (Biofast, Cat. 04020001), and DNA purity was assessed by ensuring an absorption ratio of 1.6–2.0 at 260/280 nm.

### Inclusion and exclusion criteria of clinical samples

#### Clinical samples were collected from the following participants:

1. Individuals suspected of having fragile X syndrome or unexplained intellectual disability, characterized by intellectual disability, developmental delays, behavioral signs (e.g., long face, large ears, attention deficit hyperactivity disorder, autism-like behavior), or other indicative features.
2. Patients with menstrual disorders, premature ovarian failure, ovarian hypoplasia, unexplained primary or secondary infertility, or those preparing for in vitro fertilization (IVF) who require ovarian function assessment.
3. Individuals with a family history of the conditions: females with a history of spontaneous abortion or abortion due to fetal growth retardation; Individuals seeking genetic counseling.

#### Clincial samples were excluded if with the following conditions :

1. Incomplete clinical information.
2. Sample aggregation or clotting.
3. Severe hemolysis.
4. Duplicate samples.
5. Samples deemed unsuitable for testing by the investigator.
6. Samples where the entire test could not be completed due to instrumental or human factors (e.g., sample contamination during processing).
7. Samples lacking a reference reagent result.

### Detecting methods

#### Four-primer *FMR1* PCR/CE assay

**Fig. 2:**
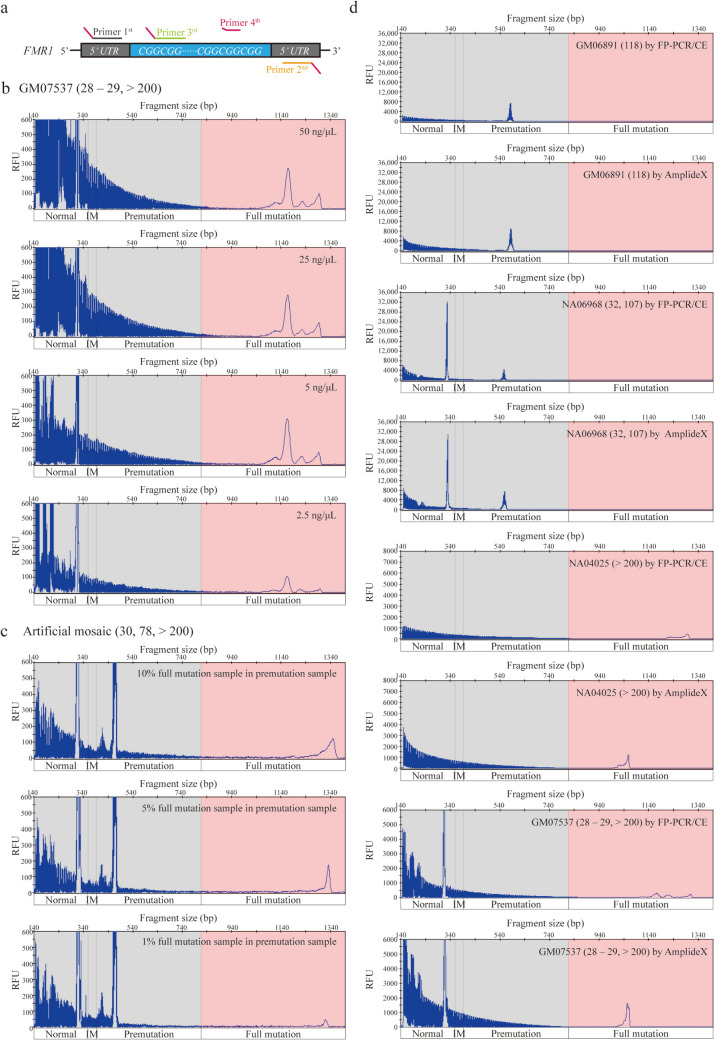
Primer design and analytical validation of the FP-PCR/CE assay. **a** Design of four primers for the assay; **b** Detection of serial dilutions of sample GM07537 (28–29, > 200) at concentrations of 50, 25, 5 and 2.5 ng/μL; **c** Detection of FM sample NA06897 (> 200) mixed into the background of NA06894 (30, 78) at mass fractions of 10%, 5%, and 1%; **d** Concordance between FP-PCR/CE and AmplideX in detecting the following samples: GM06891 (118), NA06968 (32, 107), NA04025 (> 200), and GM07537 (28–29, > 200). *IM* intermediate, *RFU* relative fluorescence units

This FP-PCR/CE assay combines four-primer fluorescent PCR with CE for *FMR1* allele analysis. In this technology, the first primer comprises the upstream sequence of the CGG repeat region; the second primer comprises the downstream sequence of the CGG repeat region; the third primer comprises the CGG repeat region; and the fourth primer comprises a non-human sequence, which is complementary to the 5’-end of the first, second and third primers (Fig. 2a and Supplemental Table 1).

**Table 1:** Detailed information of 1690 participants

Clinical diagnosis	Enrolled participants			
	*N*	Gender		Median age/y (Min–Max)
		M	F	
Fragile X syndrome/Fragile X chromosome/ *FMR1* gene full mutation	53	52	1	6.50 (1.25–21.00)
Behavioral/verbal/comprehensive developmental delay/retardation/disability	335	272	63	3.75 (1.00–31.00)
Autism spectrum disorders/socialization disorders	243	215	28	4.08 (1.33–31.00)
Intellectual disability/hypogonadism/learning disabilities	91	71	20	8.00 (2.67–40.00)
Attention deficit and hyperactivity disorder	42	30	12	8.00 (3.58–12.58)
Family history of related diseases	187	65	122	33.58 (1.00–88.00)
Ovarian regression/failure/premature aging/menstrual disorders	50	0	50	30.5 (20.00–41.00)
Miscarriage pending investigation/recurrent spontaneous abortion	28	0	28	30.5 (23.00–39.00)
Infertility/secondary infertility/primary infertility	504	2	502	31.00 (21.00–46.00)
Genetic counseling/other	157	10	147	32.00 (2.00–51.00)
Total	1690	717	973	25.00 (1.00–88.00)

*N* number, *M* male, *F* female, *y* year

In the four-primer PCR process, extracted DNA samples were subjected to amplification under the following cycling conditions: initial denaturation at 95 °C for 5 min; 10 cycles of 95 °C for 35 s, 62 °C for 35 s, and 68 °C for 2 min; followed by 20 cycles of 95 °C for 35 s, 62 °C for 35 s, 68 °C for 2 min (+ 20 seconds per cycle); and a final extension at 72 °C for 10 min. Following the PCR, the samples were prepared for CE analysis. A 0.5 μL of aliquot of each PCR product was mixed with 10 μL of a 3% GeneScan 1200 LIZ Size Standard (4,379,950, Applied Biosystems). The mixture was denatured at 95°C for 3 min, then cooled on ice for 2 min before analysis on a genetic analyzer (3500Dx, Applied Biosystems). The CE analysis was performed using the following parameters: 50 cm capillary (4,404,685, Applied Biosystems), PoP-7 Polymer (4,393,708, Applied Biosystems), injection voltage of 1.6 kV, injection time of 20 s, run voltage of 15 kV, and run time of 2700 s. Upon completion, CGG repeat sizing was analyzed using GeneMapper 5.0 or BioFast GenoAnalyzer using a reference-based standard curve.

#### AmplideX *FMR1* PCR/CE assay

AmplideX comprises two types of assays: the CGG RP PCR assay and the Gene-specific PCR assay (excluding CGG-specific primers). All assays were performed according to the standard protocol provided with the AmplideX *FMR1* PCR/CE Reagents Kit. CGG repeat sizing was analyzed using GeneMapper 5.0 software. All experiments in this study used the CGG RP PCR assay, unless otherwise indicated.

#### Southern blot

Genomic DNA was digested with *EagI* and *EcoRI* in CutSmart buffer, and the Southern blot assay was performed as previously described [24]. In samples with normal *FMR1* alleles, the assay produced a 2.8 kb band. In contrast, samples with FM alleles generated a band of ≥ 5.2 kb.

### Limit of detection

#### DNA input

Serial dilutions of Coriell DNA samples (NA13537, NA20232, GM20230, NA06905, NA06968, NA06892, GM06891, GM07537, NA04025, NA06897) were prepared in TE buffer at concentrations of 50, 25, 5, and 2.5 ng/μL. The DNA input limit of detection was defined as the lowest concentration that consistently yielded reliable results across triplicate analyses.

#### Mosaicism

FM Coriell DNA standard (NA06897) was mixed into a background of NOR or PM standards (NA18073, NA06894, GM06891) at mass fractions of 10%, 5%, and 1%, where mass fraction is defined as the ratio of the mass of the full-mutation allele to the total DNA mass in the mixture. The mosaicism limit of detection was defined as the lowest fraction that yielded consistent results across triplicate analyses.

### Statistical analysis

#### CGG repeat sizing analysis

Sizing results were interpreted based on the American College of Medical Genetics and Genomics (ACMG) grading criteria [25]. A result was considered consistent if it fell within ± 5 CGG repeats for alleles with fewer than 55 repeats, ± 10 repeats for alleles with 56 to 100 repeats, or within ± 2 standard deviations for alleles with more than 100 repeats. Discrepancies exceeding these thresholds were classified as inconsistent.

#### Consistency analysis

Consistency analysis utilized Cohen’s kappa coefficient to assess agreement between assays using the Landis-Koch criteria: κ ≥ 0.75 indicates ‘excellent’ agreement; 0.40 ≤ κ < 0.75 represents ‘moderate to good’ agreement; and κ < 0.40 suggests ‘poor’ agreement. The kappa statistics were calculated as follows:

$$\upkappa =\frac{{P}_{0}-{P}_{e}}{1-{P}_{e}}$$

where:

*P*_*0*_ is the agreement proportion observed in our data, and,

*Pₑ* is the agreement proportion that may be expected by mere chance.

#### Clinical sample size calculation

To determine the minimum sample size required to achieve minimum statistically significant results, we performed sample size calculation using the formula:

$$n=\frac{{\left[{z}_{1-\frac{\alpha }{2}}\right]}^{2}\cdot p\left(1-p\right)}{{\delta }^{2}}$$

With expected sensitivity = 95% ($p$ = 0.95), specificity = 90% ($p$ = 0.90), allowable error *δ* = 5%, and *α* = 0.05 ($z$ = 1.96), the minimum required sample size was 73 positive and 139 negative cases.

#### Quality control

The study adhered to GCP (Good Clinical Practice) standards for robust quality control [26].

## Results

### Analytical performance of FP-PCR/CE in CGG repeat quantification

FP-PCR/CE assay can accurately identify and size *FMR1* alleles (no less than 200 CGG repeats), and detect low abundance full mutation size mosaics with up to at least 1,300 CGG repeats. To validate the accuracy of the FP-PCR/CE in detecting *FMR1* alleles, we analyzed three internationally recognized *FMR1* DNA standard sets. The FP-PCR/CE demonstrated 100% concordance with the expected number of *FMR1* alleles and their CGG repeat sizes across WHO, NIFDC, and Coriell DNA standards, including accurate detection of alleles with 87 and > 200 CGG repeats in NIFDC mosaic samples P11/P12 (Supplemental Tables 2–4). Limit of detection testing showed consistent performance of FP-PCR/CE at DNA input levels of 50, 25, 5, and 2.5 ng/μL (Fig. 2b). FP-PCR/CE also successfully identified mosaicism at a 1% mass fraction (Fig. 2c). Comparative analysis using Coriell DNA standards demonstrated 100% genotype concordance between AmplideX and FP-PCR/CE (Fig. 2d and Supplemental Table 4). Results were further validated by Southern blot analysis, which confirmed consistent identification of both PM and FM alleles (Supplemental Fig. 1). The FP-PCR/CE assay also demonstrated significant operational advantages, reducing total testing time to under 7 h and lowering testing costs to below $80 per test.

### Clinical validation of FP-PCR/CE for *FMR1* allele characterization.

To validate the clinical performance of FP-PCR/CE, a total of 1717 participants were initially enrolled across six centers. After excluding cases due to consent withdrawal (*N* = 20), incomplete sample collection (*N* = 2), inadequate sample quality (*N* = 2), insufficient sample volume (*N* = 2), and duplicate enrollment (*N* = 1), 1690 participants were included in the final analysis (Fig. 1). The cohort consisted of 717 males and 973 females, with an age range of 1 to 88 years (median: 25 years). Demographic and clinical characteristics are detailed in Table 1.

All 1690 clinical samples were analyzed for *FMR1* alleles in parallel using FP-PCR/CE and AmplideX assays. The two methods showed complete concordance for *FMR1* allele characterization (κ = 1.0; Table 2). The results included 1519 samples (89.88%) with NOR alleles, 9 (0.53%) as IM, 46 (2.73%) as PM alleles, and 116 (6.86%) as FM alleles. For further verification, all PM/FM cases (*N* = 162), along with 81 randomly selected cases with NOR/IM alleles, were reanalyzed via Southern blot. FP-PCR/CE again achieved 100% concordance with Southern blot results (κ = 1.0; Supplemental Table 5). This independent verification reinforces FP-PCR/CE’s established clinical reliability for *FMR1* allele characterization, consistent with previous validation studies.

**Table 2:** Concordance of FP-PCR/CE and AmplideX in detecting 1690 clinical samples

FP-PCR/CE	AmplideX				*N*	Agreement% (95% CI)	Kappa
	NOR	IM	PM	FM			
NOR	1519	0	0	0	1519	100% (0.9975, 1)	1
IM	0	9	0	0	9	100% (0.7008, 1)	1
PM	0	0	46	0	46	100% (0.9229, 1)	1
FM	0	0	0	116	116	100% (0.9679, 1)	1
Total	1519	9	46	116	1690	100% (0.9977, 1)	1

*FP-PCR/CE* four-primer *FMR1* PCR/capillary electrophoresis assay,* N* number, *NOR* normal, *IM* intermediate, *PM* premutation, *FM* full mutation

### Sensitivity of FP-PCR/CE in detecting mosaicism

**Fig. 3:**
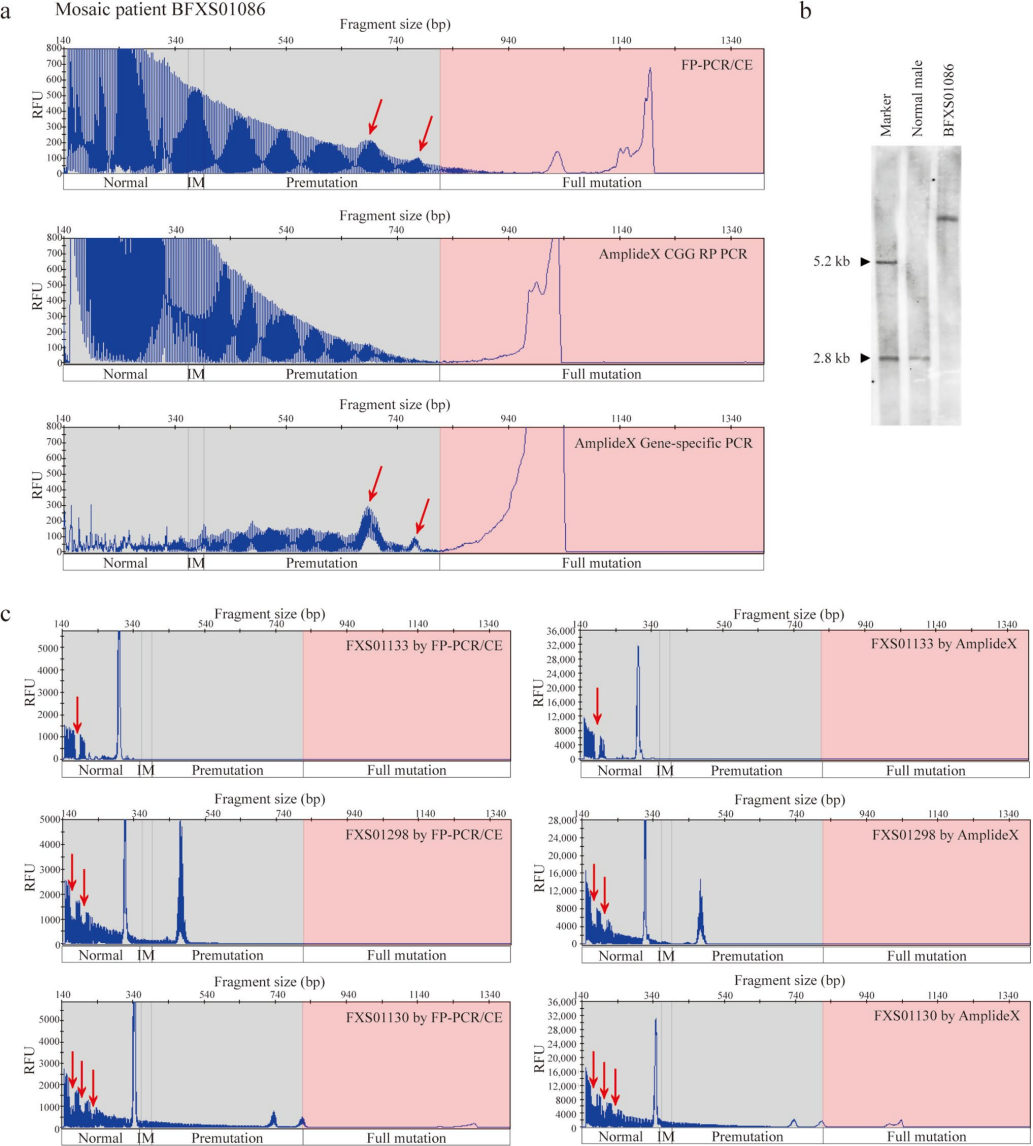
Clinical performance of the FP-PCR/CE compared to AmplideX and Southern blot. **a** Detection of a mosaic participant (BFXS01086) by FP-PCR/CE, AmplideX CGG RP PCR and AmplideX Gene-specific PCR. The arrowhead highlighted the mosaic peak; **b** Southern blot results from a normal male and the mosaic participant (BFXS01086); **c** Detection of AGG interruptions in participants FXS01133/FXS01298/FXS01130 by FP-PCR/CE and AmplideX. Arrowheads indicated the location of AGG interruptions. *IM* intermediate, *RFU* relative fluorescence units

Among the 116 FM clinical samples, CGG repeat size mosaics were assessed using FP-PCR/CE and AmplideX. While AmplideX identified mosaicism in 46 samples, FP-PCR/CE detected mosaicism in 59 cases, capturing 13 additional cases (Supplemental Table 6). Among the 46 overlapping samples, 7 showed discrepancies in peak number, with FP-PCR/CE detecting more distinct peaks. To investigate 20 discordant cases, 13 (7 were excluded due to insufficient DNA) were reanalyzed using AmplideX Gene-specific PCR (excluding CGG-specific primers). All 13 results fully matched the original FP-PCR/CE mosaic profiles (Table 3 and Fig. 3a), suggesting AmplideX CGG RP PCR may have stutter artifact interference. Additionally, Southern blot failed to detect mosaicism in these samples (Fig. 3b).

**Table 3:** Differentiation between FP-PCR/CE and AmplideX in detecting mosaicism

Clinical samples	CGG repeats		
	FP-PCR/CE	AmplideX (CGG RP PCR)	AmplideX (gene-specific PCR)
FXS01009	30, 111, > 200	> 200	30, 110, > 200
FXS01020	189, > 200	> 200	190, > 200
FXS01090	68, > 200	> 200	67, > 200
FXS01172	137, 152, > 200	> 200	136, 151, > 200
FXS01175	190, > 200	> 200	186, > 200
BFXS01073	76, > 200	> 200	75, > 200
BFXS01086	157, 187, > 200	> 200	154, 183, > 200
FXS02021	145, > 200	> 200	NA^a^
FXS02029	42, > 200	> 200	NA^a^
FXS03035	28, 52, > 200	> 200	NA^a^
FXS04017	30, 113, > 200	> 200	NA^a^
BFXS02062	105, > 200	> 200	105, > 200
BFXS03031	108, > 200	> 200	108, > 200
FXS01023	29, 188, 196, > 200	28, 194, > 200	NA^a^
FXS01051	150, 165, > 200	163, > 200	147, 162, > 200
FXS01052	103, 183, 190, > 200	189, > 200	101, 181, 188, > 200
FXS01294	42, 196, > 200	42, > 200	NA^a^
BFXS01019	103, 147, > 200	146, > 200	102, 145, > 200
FXS02035	60, 84, > 200	84, > 200	NA^a^
BFXS02253	64, 112, > 200	112, > 200	63, 111, > 200

*FP-PCR/CE* four-primer *FMR1* PCR/capillary electrophoresis assay, ^a^ *NA* not available, as samples could not be retested due to insufficient sample quantity

### FP-PCR/CE’s capability in AGG interruption detection.

To evaluate AGG interruptions, which are critical for stabilizing CGG PM alleles [27], FP-PCR/CE was applied to 25 FXS pedigrees (*N* = 122; Supplemental Table 7). FP-PCR/CE showed equivalent performance to AmplideX in identifying AGG patterns (Fig. 3c). In two multigenerational pedigrees, FP-PCR/CE revealed expected *FMR1* transmission patterns (Supplemental Fig. 2): male carriers transmitted PM alleles stably to their daughters, while female carriers exhibited a high rate of CGG expansion to full mutations in their offspring. Notably, all 24 parental PM alleles lacking AGG interruptions exhibited intergenerational CGG expansion, with 87.5% (21/24) progressing to full mutations (Supplemental Table 8).

## Discussion

In this study, we introduce FP-PCR/CE, a novel fluorescent PCR/CE assay that demonstrates superior accuracy and sensitivity in detecting *FMR1* CGG repeat expansion and mosaicism. The assay offers three key improvements over existing methods: (1) high analytical sensitivity with reliable detection from low DNA input (≥ 2.5 ng/μL), which is notably lower than the input required by AmplideX (10 ng/μL) and Southern blot (5 μg); (2) robust performance in mosaicism detection (≥ 1% mass fraction), superior to AmplideX’s detection limit of 5% [21], and (3) significantly improved operational efficiency, requiring approximately 7 h from PCR to data analysis, compared to 10 h for AmplideX and 3–5 days for Southern blot [28]. Additionally, the FP-PCR/CE assay reduces per-test costs by approximately 20% relative to AmplideX (~ $100 per assay) [29, 30].

The superiority of this assay stems from a carefully optimized four-primer system: The first and second primers respectively target the upstream and downstream sequences of the CGG repeat region, amplifying the *FMR1* 5’ UTR fragment. The third primer, which randomly binds to the CGG repeat region, generates CGG stutter peaks in 3 bp increments, allowing tracking of every single mutational allele across the CGG repeat region. The fourth primer binds to the other three primers, ensuring balanced amplification of both the *FMR1* 5’ UTR product and CGG stutter peak. Primer concentrations were also carefully optimized to achieve this balance. The concentration of the third primer in the PCR reagents is at least 1000-fold lower than the others (Supplemental Fig. 3), reducing interference from stutter peaks with mutation peaks and increasing sensitivity for long CGG repeats. This careful balance minimizes false-positive and false-negative rates.

Through optimized primer design, FP-PCR/CE identified 13 additional mosaic cases (59/116) compared to AmplideX CGG RP-PCR assay (46/116). We then reanalyzed these 13 cases using the AmplideX Gene-specific PCR, and the mosaicism results fully matched those obtained with FP-PCR/CE. This highlighted the limitation of AmplideX in detecting low-level mosaicism. Follow-up analyses of discordant cases confirmed that CGG-specific primers used in AmplideX likely generated high stutter peaks that mask the peak signal of low-level mosaicism. Recent studies have demonstrated that both size and methylation mosaicism can significantly impact clinical outcomes, with mosaic individuals often displaying milder cognitive and behavioral features compared to those with uniform full mutations [31]. Moreover, tissue mosaicism, such as between trophoblasts and somatic cells in chorionic villus sampling and between blood and skin, have been reported, though rarely. Such variability may lead to inaccurate diagnosis and missed insights into reproductive risk, adding challenges to genetic counseling [25, 32–34]. These findings highlight the need for more sensitive and accurate methodologies. FP-PCR/CE has lowered the detection limit of mosaicism to approximately 1%, offering a practical and cost-effective solution for clinical adoption.

Pedigree analysis of probands has revealed that many family members are carriers, underscoring the importance of family-based screening programs to significantly reduce the incidence of FXS [22, 25, 35]. Currently, *FMR1* gene testing is primarily based on blood samples, which are invasive and time-consuming and logistically difficult to obtain from relatives. Saliva offers a less invasive, easy-to-store, and self-collectable alternative; however, its relatively low DNA content requires assays with high analytical sensitivity [36, 37]. Our results demonstrate that FP-PCR/CE maintains robust performance with DNA inputs as low as ≥ 2.5 ng/μL, making saliva-based testing both feasible and reliable. This low DNA input requirement also expands the utility of FP-PCR/CE to preimplantation genetic testing. In assisted reproduction, DNA extracted from embryo culture supernatant, typically available in low quantities, can be used for *FMR1* testing for mutation carriers. This non-invasive approach avoids embryo biopsy, preventing the potential damage to the embryo, and potentially enhancing the success rate of in vitro fertilization. Additionally, it may decrease the risks associated with multiple pregnancies and the transmission of genetic diseases [38]. The research team has preliminarily confirmed the feasibility of using FP-PCR/CE assay in detecting *FMR1* mutation alleles in both saliva and embryo culture supernatant and plans to validate this approach in future studies.

This FP-PCR/CE is the first auxiliary diagnostic assay for FXS approved by the China National Medical Products Administration (NMPA, Registration No. 20243400096). However, certain limitations remain. Rare variants in the *FMR1* primer-binding regions may affect amplification and result interpretation, requiring secondary confirmation. Moreover, the current version of FP-PCR/CE does not assess *FMR1* methylation status, which is for predicting clinical outcomes among PM and FM carriers [39, 40]. Future enhancements of this assay integrating *FMR1* methylation analysis for a more comprehensive diagnosis are currently under validation. Furthermore, this four-primer technology is a versatile method that can be adapted to a range of repeat expansion disorders. It has already demonstrated progress in the diagnosis of diseases such as myotonic dystrophy type 1/2, spinocerebellar ataxia, Fuchs’ endothelial corneal dystrophy, and neuronal intranuclear inclusion disease. We believe this technology provides an efficient and accurate platform that holds important implications for broader disease diagnosis and clinical management.

## Supplementary Information

Below is the link to the electronic supplementary material.

Supplementary file 1 (PPTX 15926 KB)

Supplementary file 2 (DOCX 64 KB)

## Data Availability

The data that support the findings of this study are available from the corresponding author upon reasonable request.
