# Supplementary material for: Enhanced accuracy and sensitivity in detecting FMR1 CGG repeats: a multicenter evaluation of a novel PCR-capillary electrophoresis assay
Source: World J Pediatr. 2025 Sep 12;21(10):1040–52. doi: 10.1007/s12519-025-00977-5 (PMC12578688; doi:10.1007/s12519-025-00977-5)
Supplement: Supplementary file 1 — (PPTX 15926 KB) [file 12519_2025_977_MOESM1_ESM.pptx]

## Slide 1
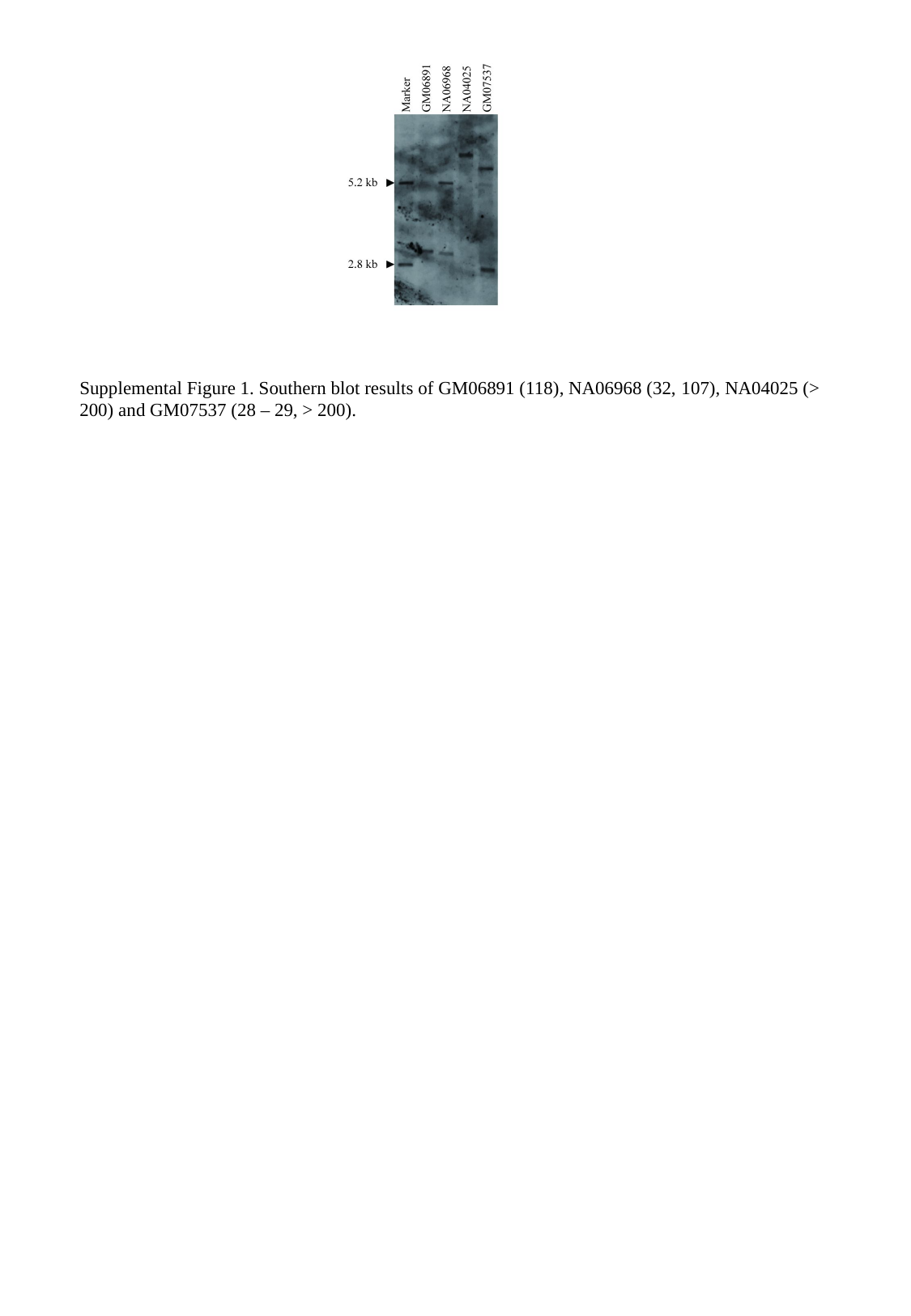

Supplemental Figure 1. Southern blot results of GM06891 (118), NA06968 (32, 107), NA04025 (> 200) and GM07537 (28 – 29, > 200).

## Slide 2
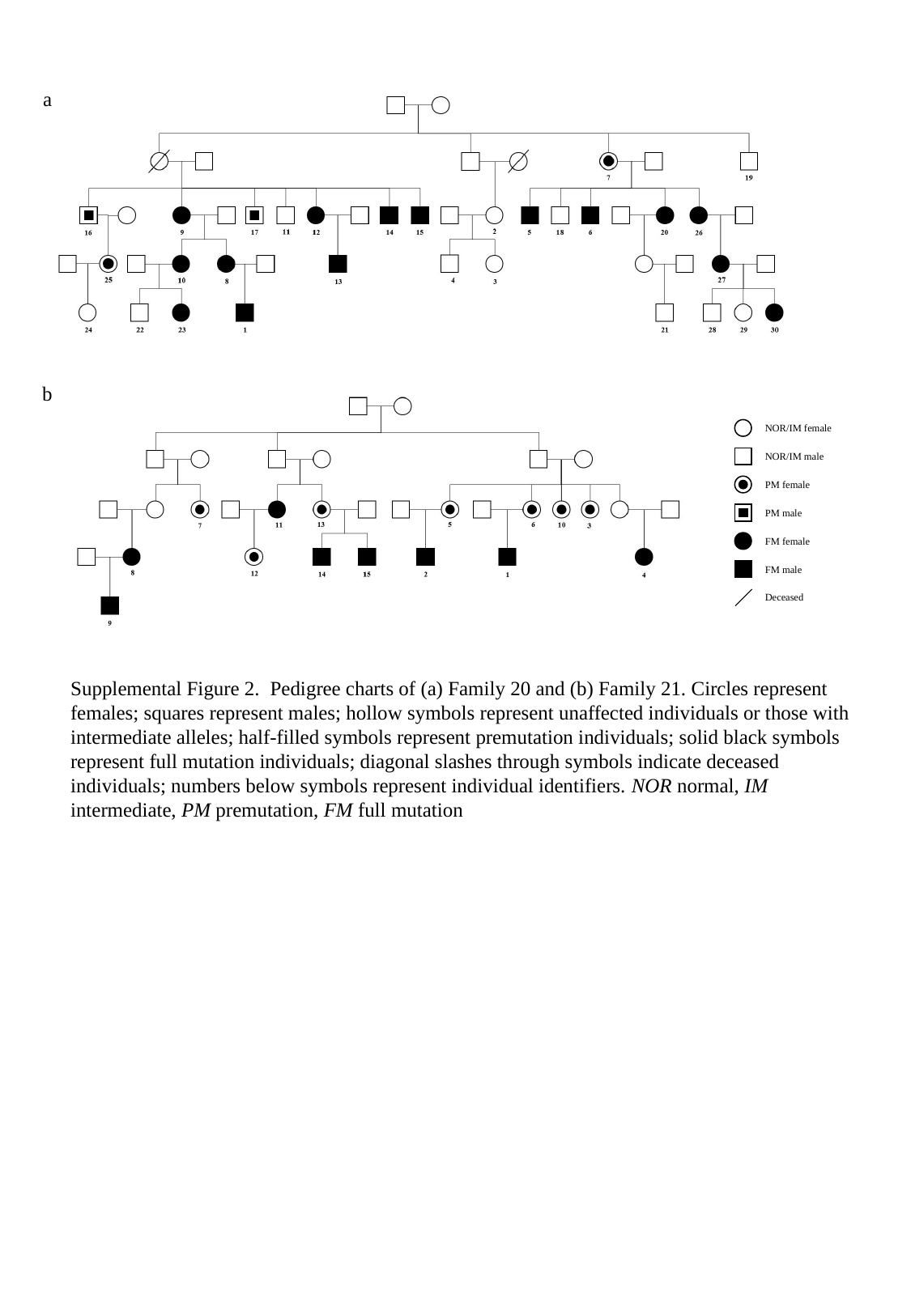

a
b
NOR/IM female
NOR/IM male
PM female
PM male
FM female
FM male
Deceased
Supplemental Figure 2. Pedigree charts of (a) Family 20 and (b) Family 21. Circles represent females; squares represent males; hollow symbols represent unaffected individuals or those with intermediate alleles; half-filled symbols represent premutation individuals; solid black symbols represent full mutation individuals; diagonal slashes through symbols indicate deceased individuals; numbers below symbols represent individual identifiers. NOR normal, IM intermediate, PM premutation, FM full mutation

## Slide 3
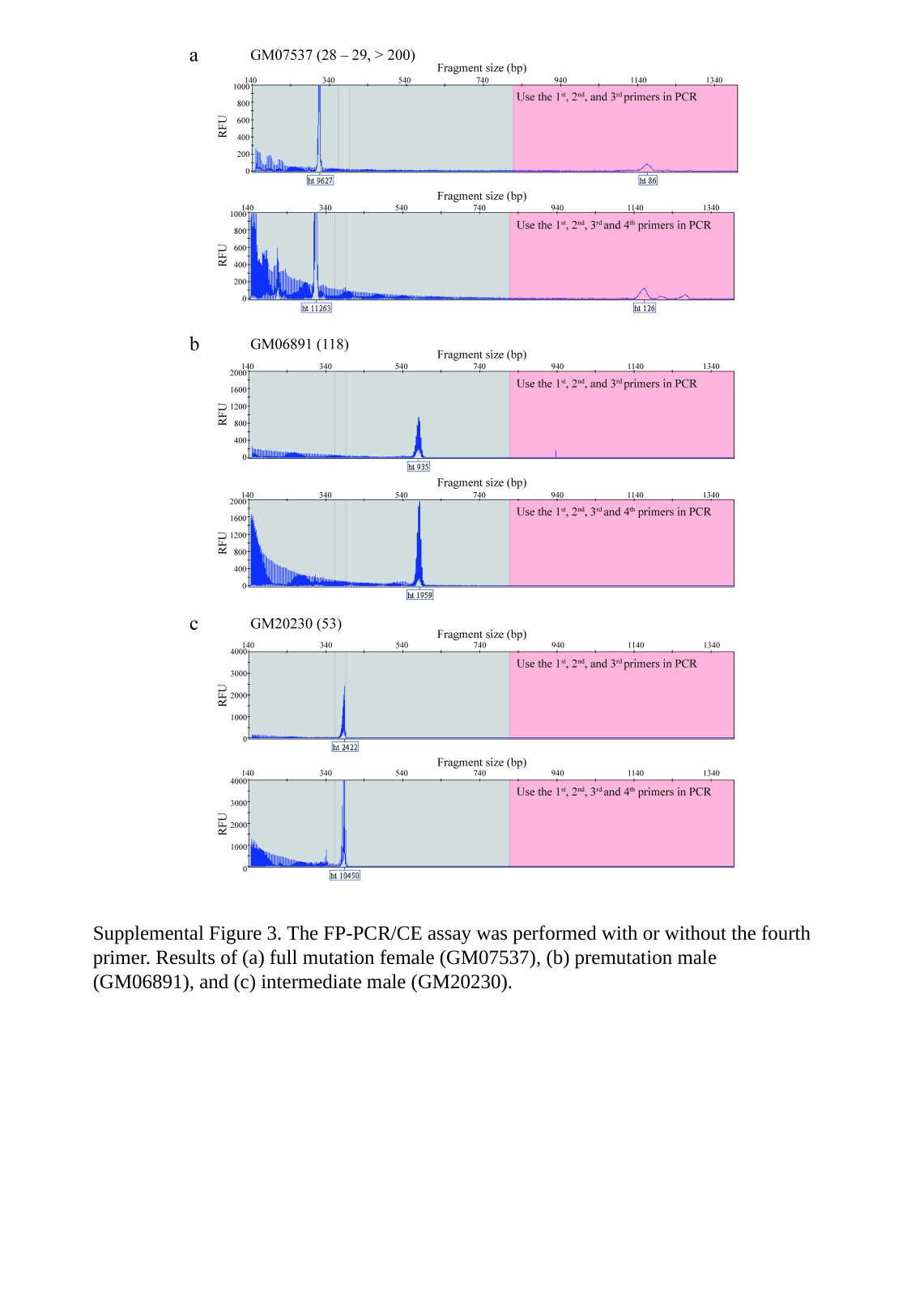

Supplemental Figure 3. The FP-PCR/CE assay was performed with or without the fourth primer. Results of (a) full mutation female (GM07537), (b) premutation male (GM06891), and (c) intermediate male (GM20230).​
