# Supplementary material for: Enhanced accuracy and sensitivity in detecting FMR1 CGG repeats: a multicenter evaluation of a novel PCR-capillary electrophoresis assay
Source: World J Pediatr. 2025 Sep 12;21(10):1040–52. doi: 10.1007/s12519-025-00977-5 (PMC12578688; doi:10.1007/s12519-025-00977-5)
Supplement: Supplementary file 2 — (DOCX 64 KB) [file 12519_2025_977_MOESM2_ESM.docx]

Supplemental Table 1. The polynucleotide sequences of the PCR primers.

| Primer | Polynucleotide sequence  (from the 5’-end to the 3’-end) |
| --- | --- |
|  |  |
| 1^st^ | GCACGCTCCTGCACAGCCTCCTCAGCTCCGTTTCGGTTTCACTTCC |
| 2^nd^ | GCACGCTCCTGCACAGCCTCAGCGCCATTGGAGCCCCGCACTTCCA |
| 3^rd^ | CTCGACGCACGCTCCTGCACAGCCTCCGGCGGCGGCGGCGG |
| 4^th^ | CTCGACGCACGCTCCTGCACAGCCTC |

Supplemental Table 2. Concordance with the expected CGG repeat size in the WHO standards.

| Sample No. | Allele | CGG repeats | |
| --- | --- | --- | --- |
|  |  | WHO | FP-PCR/CE |
| 07/120 | NOR | 19 – 24, 28 – 33 | 22, 31 |
| 07/122 | PM | 30 – 36, 100 – 132 | 34, 90, 106, 117 |
| 07/168 | FM | 33 – 41, 300 – 401 | 39, > 200 |
| 07/170 | FM | 353 – 960 | > 200 |
| 07/174 | PM | 97 – 127 | 76, 89, 98, 113, 121 |

*NOR* normal, *PM* premutation, *FM* full mutation

Supplemental Table 3. Concordance with the expected CGG-repeat size in the NIFDC standards.

| Sample No. | Allele | CGG repeats | |
| --- | --- | --- | --- |
|  |  | NIFDC | FP-PCR/CE |
| P1 | IM | 46 ± 1 | 46 |
| P2 | IM | 54 | 54 |
| P3 | PM | 30 ± 1, 69 ± 3 | 30, 67 |
| P4 | PM | 29 ± 1, 155 ± 5 | 30, 156 |
| P5 | PM | 56 ± 1 | 55 |
| P6 | PM | 100 ± 5 | 100 |
| P7 | FM | 30 ± 1, > 200 | 29, > 200 |
| P8 | FM | 29 ± 1, > 200 | 29, > 200 |
| P9 | FM | > 200 | > 200 |
| P10 | FM | > 200 | > 200 |
| P11 | PM and FM mosaic | 86 ± 5, > 200 | 87, > 200 |
| P12 | NOR and FM mosaic | 13 ± 1, > 200 | 12, > 200 |
| N1 | NOR | 29 ± 1 | 29 |
| N2 | NOR | 31 ± 1, 36 ± 1 | 31, 36 |
| N3 | NOR | 29 ± 1, 30 ± 1 | 29, 30 |
| N4 | NOR | 30 ± 1, 34 ± 1 | 29, 34 |
| N5 | NOR | 30 ± 1 | 30 |
| N6 | NOR | 36 ± 1 | 36 |
| N7 | Non-human DNA | None | None |
| N8 | Non-human DNA | None | None |
| N9 | *FMR1* deletion | None | None |

*NOR* normal, *IM* intermediate, *PM* premutation, *FM* full mutation

Supplemental Table 4. Concordance with the expected CGG repeat size in Coriell DNA.

| Coriell DNA No. | Genotype | CGG repeats | | |
| --- | --- | --- | --- | --- |
|  |  | Coriell | FP-PCR/CE | AmplideX |
| GM20230 | Intermediate male | 53 | 54 | 54 |
| NA20232 | Intermediate male | 46 | 46 | 46 |
| NA06905 | Premutation female | 23, 70 | 23, 79 | 23, 79 |
| NA06968 | Premutation female | 32, 107 | 33, 112 | 33, 110 |
| NA06892 | Premutation male | 93 | 93 | 91 |
| GM06891 | Premutation male | 118 | 120 | 119 |
| GM07537 | Full mutation female | 28-29, > 200 | 29, > 200 | 29, > 200 |
| NA04025 | Full mutation male | > 200 | > 200 | > 200 |
| NA06897 | Full mutation male | > 200 | > 200 | > 200 |

Supplemental Table 5. The concordance of 243 selected clinical samples between FP-PCR/CE and Southern blot assays.

| FP-PCR/CE | Southern blot | | | | *N* | Agreement %（95% CI） | Kappa |
| --- | --- | --- | --- | --- | --- | --- | --- |
|  | NOR | IM | PM | FM |  |  |  |
| NOR | 81 | 0 | 0 | 0 | 80 | 100% (0.9547, 1) | 1 |
| IM | 0 | 1 | 0 | 0 | 1 | 100% (0.2065, 1) | 1 |
| PM | 0 | 0 | 46 | 0 | 46 | 100% (0.9229, 1) | 1 |
| FM | 0 | 0 | 0 | 69 | 69 | 100% (0.9473, 1) | 1 |
| Total | 80 | 1 | 46 | 69 | 243 | 100% (0.9844, 1) | 1 |

*NOR* normal, *IM* intermediate, *PM* premutation, *FM* full mutation

Supplemental Table 6. Mosaicism detected by FP-PCR/CE and AmplideX in full mutation samples.

| Gender | *N* | FP-PCR/CE | | AmplideX | |
| --- | --- | --- | --- | --- | --- |
|  |  | Mosaic | Non-mosaic | Mosaic | Non-mosaic |
| Male | 85 | 39 | 46 | 26 | 59 |
| Female | 31 | 20 | 11 | 20 | 11 |
| Total | 116 | 59 | 57 | 46 | 70 |
| Mosaicism rate | NA | 51% | NA | 40% | NA |

*FM* full mutation, *NA* not applicable

Supplemental Table 7. Characteristics of FXS pedigrees.

| Member | Sample | Gender | CGG repeats | | Allele | AGG interruptions | |
| --- | --- | --- | --- | --- | --- | --- | --- |
|  |  |  | FP-PCR/CE | AmplideX |  | FP-PCR/CE | AmplideX |
| Family 1 |  |  |  |  |  |  |  |
| Mother | FXS01005 | F | 30, 76 | 30, 76 | PM | 2 | 2 |
| Proband | FXS01006 | M | > 200 | > 200 | FM | 0 | 0 |
| Family 2 |  |  |  |  |  |  |  |
| Proband | FXS01009 | M | 30, 111, > 200 | > 200 | FM | 0 | 0 |
| Mother | FXS01010 | F | 29, 143, > 200 | 29, 142, > 200 | FM | 2 | 2 |
| Family 3 |  |  |  |  |  |  |  |
| Proband | FXS01020 | M | 189, > 200 | > 200 | FM | 0 | 0 |
| Mother | FXS01021 | F | 36, 93, 96 | 36, 91, 95 | PM | 2 | 2 |
| Family 4 |  |  |  |  |  |  |  |
| Proband | FXS01022 | M | > 200 | > 200 | FM | 0 | 0 |
| Mother | FXS01023 | F | 29, 188, 196, > 200 | 29, 193, > 200 | FM | 2 | 2 |
| Aunt | FXS01024 | F | 29, 185, > 200 | 28, 183, > 200 | FM | 2 | 2 |
| Uncle | FXS01031 | M | > 200 | > 200 | FM | 0 | 0 |
| Grandmother | FXS01032 | F | 29, 86 | 29, 86 | PM | 2 | 2 |
| Grandfather | FXS01033 | M | 29 | 29 | NOR | 2 | 2 |
| Great grandmother | FXS01034 | F | 39, 71 | 39, 71 | PM | 3 | 3 |
| Family 5 |  |  |  |  |  |  |  |
| Proband | FXS01027 | M | 98, > 200 | 97, > 200 | FM | 0 | 0 |
| Sister | FXS01028 | F | 29, > 200 | 29, > 200 | FM | 2 | 2 |
| Mother | FXS01029 | F | 30, > 200 | 30, > 200 | FM | 2 | 2 |
| Family 6 |  |  |  |  |  |  |  |
| Proband | FXS01052 | M | 103, 183, 190, > 200 | 189, > 200 | FM | 0 | 0 |
| Grandfather | FXS01053 | M | 30 | 30 | NOR | 2 | 2 |
| Grandmother | FXS01054 | F | 30, 75 | 30, 75 | PM | 2 | 2 |
| Family 7 |  |  |  |  |  |  |  |
| Proband | FXS01090 | M | 68, > 200 | > 200 | FM | 0 | 0 |
| Mother | FXS01130 | F | 39, 173, 200, > 200 | 39, 171, 197, > 200 | FM | 3 | 3 |
| Aunt | FXS01131 | F | 36, 39 | 36, 39 | NOR | 2 | 2 |
| Sister | FXS01132 | F | 29, > 200 | 29, > 200 | FM | 2 | 2 |
| Family 8 |  |  |  |  |  |  |  |
| Proband | FXS01104 | F | 30, > 200 | 30, > 200 | FM | 2 | 2 |
| Mother | FXS01105 | F | 18, 30, 127, 153 | 30, 126, 152 | PM | 2 | 2 |
| Counsin-1 | FXS01106 | M | 116, > 200 | 115, > 200 | FM | 0 | 0 |
| Aunt-1 | FXS01107 | F | 29, 127, 150 | 29, 126, 147 | PM | 2 | 2 |
| Counsin-2 | FXS01108 | M | 49, 140, > 200 | 49, 140, > 200 | FM | 0 | 0 |
| Aunt-2 | FXS01109 | F | 29, 147, > 200 | 29, 146, > 200 | FM | 2 | 2 |
| Family 9 |  |  |  |  |  |  |  |
| Proband | FXS01051 | M | 150, 165, > 200 | 163, > 200 | FM | 0 | 0 |
| Brother | FXS01146 | M | 36 | 36 | NOR | 3 | 3 |
| Mother | FXS01147 | F | 28, 36, > 200 | 28, 36, > 200 | FM | 3 | 3 |
| Family 10 |  |  |  |  |  |  |  |
| Proband | FXS01172 | M | 137, 152, > 200 | > 200 | FM | 0 | 0 |
| Mother | FXS01173 | F | 31, 131, 165 | 31, 130, 163 | PM | 2 | 2 |
| Aunt | FXS01174 | F | 29, 31 | 29, 31 | NOR | 2 | 2 |
| Family 11 |  |  |  |  |  |  |  |
| Proband | FXS01195 | M | > 200 | > 200 | FM | 0 | 0 |
| Mother | FXS01196 | F | 29, 97 | 29, 95 | PM | 2 | 2 |
| Family 12 |  |  |  |  |  |  |  |
| Proband | FXS01223 | M | > 200 | > 200 | FM | 0 | 0 |
| Mother | FXS01224 | F | 29, 65 | 29, 65 | PM | 2 | 2 |
| Family 13 |  |  |  |  |  |  |  |
| Proband | FXS01254 | M | > 200 | > 200 | FM | 0 | 0 |
| Mother | FXS01298 | F | 29, 82 | 29, 82 | PM | 2 | 2 |
| Family 14 |  |  |  |  |  |  |  |
| Proband | FXS01255 | M | > 200 | > 200 | FM | 0 | 0 |
| Mother | FXS01256 | F | 36, 109, 116 | 36, 108, 114 | PM | 3 | 3 |
| Sister | FXS01293 | F | 29, 110, 127, 135, > 200 | 29, 109, 125, 134, > 200 | FM | 2 | 2 |
| Cousin | FXS01294 | M | 42, 196, > 200 | 42, > 200 | FM | 0 | 0 |
| Aunt | FXS01295 | F | 36, 118, 127 | 36, 117, 125 | PM | 3 | 3 |
| Grandmother | FXS01296 | F | 29, 36 | 29, 36 | NOR | 3 | 3 |
| Family 15 |  |  |  |  |  |  |  |
| Proband | FXS01283 | M | > 200 | > 200 | FM | 0 | 0 |
| Mother | FXS01284 | F | 29, 79 | 29, 79 | PM | 2 | 2 |
| Grandmother | FXS01291 | F | 29 | 29 | NOR | 2 | 2 |
| Grandfather | FXS01292 | M | 62 | 62 | PM | 0 | 0 |
| Family 16 |  |  |  |  |  |  |  |
| Proband | FXS01288 | M | > 200 | > 200 | FM | 0 | 0 |
| Mother | FXS01289 | F | 29, 94, 99 | 29, 93, 98 | PM | 2 | 2 |
| Family 17 |  |  |  |  |  |  |  |
| Proband | FXS02003 | M | 87, > 200 | 87, > 200 | FM | 0 | 0 |
| Mother | FXS02004 | F | 29, 164, > 200 | 29, 162, > 200 | FM | 2 | 2 |
| Family 18 |  |  |  |  |  |  |  |
| Proband | FXS02005 | F | 29, 107 | 29, 106 | PM | 2 | 2 |
| Father | FXS02009 | F | 29, 68 | 29, 68 | PM | 2 | 2 |
| Grandmother | FXS02010 | M | 69, 80 | 69, 80 | PM | 0 | 0 |
| Aunt | FXS02011 | F | 39, 94, 102 | 39, 93, 101 | PM | 3 | 3 |
| Uncle | FXS02012 | M | > 200 | > 200 | FM | 0 | 0 |
| Cousin | FXS02013 | M | > 200 | > 200 | FM | 0 | 0 |
| Family 19 |  |  |  |  |  |  |  |
| Proband | FXS02007 | F | 36, 78 | 36, 78 | PM | 2 | 2 |
| Nephew | FXS02014 | M | > 200 | > 200 | FM | 0 | 0 |
| Father | FXS02015 | M | 95 | 94 | PM | 0 | 0 |
| Sister | FXS02016 | F | 29, 89 | 29, 88 | PM | 2 | 2 |
| Family 20 |  |  |  |  |  |  |  |
| Proband | FXS02017 | M | > 200 | > 200 | FM | 0 | 0 |
| Grandmother's cousin-1 | FXS02018 | F | 29 | 28 | NOR | 2 | 2 |
| Mother's cousin-1 | FXS02019 | F | 29, 30 | 29 | NOR | 2 | 2 |
| Mother's cousin-2 | FXS02020 | M | 29 | 29 | NOR | 2 | 2 |
| Grandmother's cousin-2 | FXS02021 | M | 145, > 200 | > 200 | FM | 0 | 0 |
| Grandmother's cousin-3 | FXS02022 | M | > 200 | > 200 | FM | 0 | 0 |
| Grandmother's aunt | FXS02023 | F | 30, 69 | 30, 69 | PM | 2 | 2 |
| Mother | FXS02024 | F | 36, > 200 | 36, > 200 | FM | 3 | 3 |
| Grandmother | FXS02025 | F | 36, 123, 136, > 200 | 36, 122, 134, > 200 | FM | 3 | 3 |
| Aunt | FXS02026 | F | 36, > 200 | 36, > 200 | FM | 3 | 3 |
| Grandmother's brother | FXS02027 | M | 29 | 28 | NOR | 2 | 2 |
| Grandmother's sister | FXS02028 | F | 36, 136, > 200 | 36, 135, > 200 | FM | 3 | 3 |
| Grandmother's nephew | FXS02029 | M | 42, > 200 | > 200 | FM | 0 | 0 |
| Grandmother's brother | FXS02030 | M | > 200 | > 200 | FM | 0 | 0 |
| Grandmother's brother | FXS02031 | M | 154, > 200 | 151, > 200 | FM | 0 | 0 |
| Grandmother's brother | FXS02032 | M | 67, 101 | 67, 100 | PM | 0 | 0 |
| Grandmother's brother | FXS02033 | M | 88, 94 | 87, 92 | PM | 0 | 0 |
| Grandmother's cousin-4 | FXS02338 | M | 30 | 30 | NOR | 2 | 2 |
| Grandmother's uncle | FXS02339 | M | 29 | 29 | NOR | 2 | 2 |
| FXS02023's daughter | FXS02340 | F | 34, 120, > 200 | 34, 119, > 200 | FM | 2 | 2 |
| FXS02340's grandson | FXS02341 | M | 30 | 30 | NOR | 1 | 1 |
| Cousin-1 | FXS02342 | M | 36 | 35 | NOR | 3 | 3 |
| Cousin-2 | FXS02343 | F | 30, > 200 | 30, > 200 | FM | 2 | 2 |
| FXS02345's daughter | FXS02344 | F | 29, 53 | 29, 53 | IM | 5 | 5 |
| FXS02032's daughter | FXS02345 | F | 53, 120, 141 | 53, 119, 138 | PM | 4 | 4 |
| FXS02023's daughter | FXS02346 | F | 29, 113, 131, > 200 | 29, 112, 130, > 200 | FM | 2 | 2 |
| FXS02346's daughter | FXS02347 | F | 30, > 200 | 30, > 200 | FM | 2 | 2 |
| FXS02347's son | FXS02348 | M | 30 | 30 | NOR | 2 | 2 |
| FXS02347's daughter | FXS02349 | F | 29, 30 | 29, 30 | NOR | 2 | 2 |
| FXS02347's daughter | FXS02350 | F | 29, 193, > 200 | 29, 191, > 200 | FM | 2 | 2 |
| Family 21 |  |  |  |  |  |  |  |
| Cousin-1 | FXS04001 | M | > 200 | > 200 | FM | 0 | 0 |
| Cousin-2 | FXS04002 | M | 109, > 200 | 108, > 200 | FM | 0 | 0 |
| Anut-1 | FXS04003 | F | 30, 87, 105, 112 | 29, 86, 103, 111 | PM | 2 | 2 |
| Cousin-3 | FXS04004 | F | 30, 80, > 200 | 29, 79, > 200 | FM | 2 | 2 |
| Anut-2 | FXS04005 | F | 30, 101 | 29, 99 | PM | 2 | 2 |
| Anut-3 | FXS04006 | F | 29, 91, 97 | 29, 90, 95 | PM | 2 | 2 |
| Anut-4 | FXS04007 | F | 42, 73 | 41, 72 | PM | 3 | 3 |
| Proband | FXS04008 | F | 36, > 200 | 35, > 200 | FM | 3 | 3 |
| Son | FXS04009 | M | -21, 170, > 200 | -21, 167, > 200 | FM | 0 | 0 |
| Aunt-5 | FXS04010 | F | 30, 119 | 29, 118 | PM | 2 | 2 |
| Anut-6 | FXS04013 | F | 30, 122, > 200 | 29, 121, > 200 | FM | 2 | 2 |
| Cousin-4 | FXS04014 | F | 30, 104, 109 | 29, 102, 107 | PM | 2 | 2 |
| Anut-7 | FXS04015 | F | 37, 103 | 36, 101 | PM | 3 | 3 |
| Cousin-5 | FXS04016 | M | > 200 | > 200 | FM | 0 | 0 |
| Cousin-6 | FXS04017 | M | 30, 113, > 200 | > 200 | FM | 0 | 0 |
| Family 22 |  |  |  |  |  |  |  |
| Proband | FXS04245 | M | > 200 | > 200 | FM | 0 | 0 |
| Mother | FXS04246 | F | 2, 29, 119, 132, 136, > 200 | 29, 118, 130, 136, > 200 | FM | 2 | 2 |
| Aunt | FXS04247 | F | 40, 160, > 200 | 40, 157, > 200 | FM | 3 | 3 |
| Family 23 |  |  |  |  |  |  |  |
| Mother | FXS04248 | F | 30, 75, 87 | 30, 75, 86 | PM | 2 | 2 |
| Sister | FXS04249 | F | 30, 141, 163 | 30, 140, 160 | PM | 2 | 2 |
| Proband | FXS04250 | M | > 200 | > 200 | FM | 0 | 0 |
| Family 24 |  |  |  |  |  |  |  |
| Mother | FXS03020 | F | 30, 88 | 30, 88 | PM | 2 | 2 |
| Proband | FXS03021 | M | 105, > 200 | 105, > 200 | FM | 0 | 0 |
| Family 25 |  |  |  |  |  |  |  |
| Proband | FXS03032 | F | 30, > 200 | 29, > 200 | FM | 2 | 2 |
| Aunt | FXS03033 | F | 22, 30 | 22, 29 | NOR | 2 | 2 |
| Grandmother | FXS03034 | F | 22, 71 | 22, 71 | PM | 2 | 2 |
| Son | FXS03035 | M | 28, 52, > 200 | > 200 | FM | 0 | 0 |

*NOR* normal, *IM* intermediate, *PM* premutation, *FM* full mutation, *F* female, *M* male

Supplemental Table 8. Summary of CGG repeat expansion and AGG interruptions in premutation parents and their offspring.

| Member | CGG repeats | Allele | AGG interruptions |
| --- | --- | --- | --- |
| Group 1 |  |  |  |
| Mother | 30, 76 | PM | 2 |
| Son | > 200 | FM | 0 |
| Group 2 |  |  |  |
| Mother | 36, 93, 96 | PM | 2 |
| Son | 189, > 200 | FM | 1 |
| Group 3 |  |  |  |
| Mother | 39, 71 | PM | 3 |
| Daughter | 29, 86 | PM | 2 |
| Group 4 |  |  |  |
| Mother | 29, 86 | PM | 2 |
| Father | 29 | NOR | 2 |
| Daughter | 29, 188, 196, > 200 | FM | 2 |
| Daughter | 29, 185, > 200 | FM | 2 |
| Son | > 200 | FM | 0 |
| Group 5 |  |  |  |
| Mother | 29, 127, 150 | PM | 2 |
| Son | 116, > 200 | FM | 0 |
| Group 6 |  |  |  |
| Mother | 31, 131, 165 | PM | 2 |
| Son | 137, 152, > 200 | FM | 0 |
| Group 7 |  |  |  |
| Mother | 29, 97 | PM | 2 |
| Son | > 200 | FM | 0 |
| Group 8 |  |  |  |
| Mother | 29, 65 | PM | 2 |
| Son | > 200 | FM | 0 |
| Group 9 |  |  |  |
| Mother | 29, 82 | PM | 2 |
| Son | > 200 | FM | 0 |
| Group 10 |  |  |  |
| Mother | 36, 109, 116 | PM | 3 |
| Son | > 200 | FM | 0 |
| Daughter | 29, 110, 127, 135, > 200 | FM | 2 |
| Group 11 |  |  |  |
| Mother | 36, 118, 127 | PM | 3 |
| Son | 42, 196, > 200 | FM | 0 |
| Group 12 |  |  |  |
| Mother | 29, 79 | PM | 2 |
| Son | > 200 | FM | 0 |
| Group 13 |  |  |  |
| Mother | 29, 94, 99 | PM | 2 |
| Son | > 200 | FM | 0 |
| Group 14 |  |  |  |
| Mother | 29, 68 | PM | 2 |
| Son | 69, 80 | PM | 0 |
| Son | > 200 | FM | 0 |
| Daughter | 39, 94, 102 | PM | 3 |
| Group 15 |  |  |  |
| Mother | 39, 94, 102 | PM | 3 |
| Son | > 200 | FM | 0 |
| Group 16 |  |  |  |
| Mother | 30, 69 | PM | 2 |
| Son | 145, > 200 | FM | 0 |
| Son | > 200 | FM | 0 |
| Son | 30 | NOR | 2 |
| Daughter | 29, 113, 131, > 200 | FM | 2 |
| Group 17 |  |  |  |
| Mother | 29, 91, 97 | PM | 2 |
| Son | > 200 | FM | 0 |
| Group 18 |  |  |  |
| Mother | 30, 101 | PM | 2 |
| Son | 109, > 200 | FM | 0 |
| Group 19 |  |  |  |
| Mother | 37, 103 | PM | 3 |
| Son | > 200 | FM | 0 |
| Son | 113, > 200 | FM | 0 |
| Group 20 |  |  |  |
| Mother | 30, 75, 87 | PM | 2 |
| Son | > 200 | FM | 0 |
| Group 21 |  |  |  |
| Mother | 30, 88 | PM | 2 |
| Son | 105, > 200 | FM | 0 |
| Group 22 |  |  |  |
| Mother | 22, 71 | PM | 2 |
| Daughter | 30, > 200 | FM | 2 |
| Daughter | 22, 30 | NOR | 2 |
| Group 23 |  |  |  |
| Mother | 29, 72 | PM | 1 |
| Daughter | 37, 84 | PM | 1 |
| Group 24 |  |  |  |
| Mother | 29 | NOR | 2 |
| Father | 62 | PM | 0 |
| Daughter | 29, 79 | PM | 2 |

*NOR* normal, *IM* intermediate, *PM* premutation, *FM* full mutation
